# Feasibility and Safety of Laparoscopic Radical Colectomy for T4b Colon Cancer at a University Hospital in Vietnam

**DOI:** 10.1155/2020/1762151

**Published:** 2020-11-11

**Authors:** Thinh H. Nguyen, Hung X. Tran, Truc T. Thai, Duc M. La, Huy D. Tran, Kien T. Le, Vinh T. N. Pham, An N. T. Le, Bac H. Nguyen

**Affiliations:** ^1^Department of General Surgery, University of Medicine and Pharmacy at Ho Chi Minh City, 70000, Vietnam; ^2^Department of Gastrointestinal Surgery, University Medical Center Ho Chi Minh City, 70000, Vietnam; ^3^Department of Medical Statistics and Informatics, University of Medicine and Pharmacy at Ho Chi Minh City, 70000, Vietnam

## Abstract

**Background:**

The choice of optimal treatment strategies for T4b colon cancers has still been discussed, particularly the initiation of neoadjuvant therapy or surgery. We conducted this study to evaluate the safety and feasibility of laparoscopic multivisceral resection for T4b colon cancers.

**Methods:**

We used the retrospective design to include all 43 patients with T4b colon cancer at a university hospital in Vietnam from March 2017 to March 2019. All patients were followed 30 days after the surgery, and information about the day of the first flatus, length of hospital stay, iatrogenic complications, postoperative morbidity, mortality, and adjuvant chemotherapy was collected.

**Results:**

The mean operating time was 187 minutes (ranging from 80 to 310), the mean blood loss was 64.3 ml (5-200), and the conversion rate was 2.3%. The mean number of lymph nodes harvested was 15.5 (SD = 8.06), and 33 patients (76.7%) had at least 12 lymph nodes harvested. A total of 21 patients (48.8%) had lymph node metastases with a mean number of lymph node metastases of 1.89 (SD = 3.4). The radial resection margin was R0 in all 43 patients (100%). The median time until the first flatus and hospital stay were 3 days (2–5) and 7.1 (6–11) days, respectively. There was no mortality at 30 days postoperatively, and one patient had iatrogenic complication (2.3%).

**Conclusion:**

Laparoscopic radical colectomy was feasible and safe for patients with T4b colon cancer except those requiring major and complicated reconstruction.

## 1. Introduction

Colorectal cancer (CRC) is among the most common alimentary tract cancers and is also an important cause of death worldwide. According to GLOBOCAN 2018 [1], colorectal cancer is the fourth most commonly diagnosed malignancy and the fourth leading cause of cancer death, accounting for about 1.4 million new cases and almost 700000 deaths. The incidence and mortality rate of CRC are rising rapidly in many low- and middle-income countries but are stabilizing or decreasing in developed countries [2]. In the United States, the CRC incidence rate has been declining for several decades at a stable rate of 3% annually from 2003 to 2012 [3]. In Vietnam, CRC ranks the second among alimentary tract cancers and is often diagnosed in advanced stages when the tumor is invasive, complicated, or distant metastatic [4, 5]. Among these cases, the survival rate after 5 years is low and the quality of life after surgery is not high.

Among CRC and T4b colon cancer patients, the choice of optimal treatment strategies has still been discussed, particularly the initiation of neoadjuvant therapy or surgery. On the one hand, patients with T4b colon cancer can undergo neoadjuvant therapy followed by surgery. This approach has been shown to have significant downstaging of tumor, improvement of resection ability, and better survival outcomes in esophageal cancer [6], gastric cancer [7], and rectal cancer [8, 9]. Further, other potential benefits in this approach include decimation of micrometastases and restriction of tumor cell scattering intraoperatively. In some recent studies, neoadjuvant chemotherapy for locally advanced colon cancer (LACC) can improve the survival rate as compared to surgical resection followed by adjuvant chemotherapy [10, 11]. However, this approach for T4b colon cancer is controversial. About 20% of patients initiated with neoadjuvant treatment have been reported to develop stage III or IV of upstaging events based on the American Joint Committee on Cancer (AJCC) classification (7^th^ edition) [12]. Moreover, patients are also vulnerable to adverse effects and toxicity from neoadjuvant chemotherapy. There are also potential risks of tumor growth during the preoperative treatment period which may result in bowel obstruction or perforation where emergency surgery is needed.

On the other hand, patients with T4b colon cancer can have first-line treatment with surgery, followed by adjuvant therapy [13]. This approach has been shown to be beneficial for patients who might not be able to tolerate intensive neoadjuvant treatment. Therefore, the current standard treatment for T4b colon cancer is based on traditional dogma among surgeons who trust in en bloc oncological multivisceral resection (MVR) [14–17], while adjuvant chemotherapy was recommended for patients with TNM stage III disease or those with unfavorable histopathological findings after recovery from surgery. In Vietnam, T4b cancer patients are typically old, comorbid, and often admitted to the hospital in poor health condition. Thus, those who initiated neoadjuvant treatment may result in exhaustion and financial difficulty, which in turn prevent them from undergoing further surgery.

To do MVR, a laparoscopic approach can enhance recovery after surgery. A study by Shukla et al. [18] showed that laparoscopic surgery is feasible in T4 colon cancers and suggested that laparoscopy can be considered an alternative approach for T4 colon cancers with the advantage of a higher recovery rate. Takahashi et al. [16] reviewed 84 T4b colon cancer patients who underwent laparoscopic MVR and revealed that the laparoscopy approach can be considered for surgical T4b cancers, except for urinary tract invasion patients who may require visceral reconstruction. Further, Yamanashi et al. [17] demonstrated that selected LACC patients should not be excluded from laparoscopic surgery. However, laparoscopic colectomy for T4 tumors is associated with challenging technical feasibility, high conversion rate, inadequate oncologic clearance, and short-term outcomes [17]. To date, laparoscopic surgery has been widely adopted, and new technical innovation, procedures, and evidence-based knowledge are persistently emerging [19]. The surgeon's skill and experience have been improving alongside with technological advances in manufacturing surgical energy devices and the outstanding development in imaging technology in recent years such as high-density (HD) and full HD wide view image systems [20, 21]. Many new technologies have been launching such as 3D technology, 4K image, and near-infrared imaging (NIR) [22–25]. These technologies can be integrated into the same endoscopic screen to help optimize surgical quality and improve patient safety.

In Vietnam, however, the indication of laparoscopic MVR for T4b colon cancer may require further scrutiny and debate. Vietnamese surgeons often encounter a situation where the tumor invades the abdominal wall, gonadal vessels, pancreas, stomach, spleen, and bladder through laparoscopic surgery [4, 5]. This is especially common in hospitals in rural areas where preoperative diagnostic is not effective. In such cases, it is challenging for surgeons to decide whether conversion, palliative surgery, or laparoscopic radical colectomy is required. Before 2010, patients with T4b colon cancer often underwent open colectomy at the beginning or conversion right after diagnostic laparoscopy. Since then, thanks to the improvement of both the laparoscopic system and laparoscopic surgical skills among surgeons, laparoscopic colectomy has been used more often for T4b colon cancers with the belief that this approach could enhance recovery after surgery. Recently, there are several centers in Ho Chi Minh City using this approach for patients with T4b colon tumors, but the feasibility and safety of this technique have yet been investigated. This study was aimed at evaluating the feasibility and safety of laparoscopic radical colectomy for T4b colon cancer at a university hospital in Vietnam.

## 2. Materials and Methods

### 2.1. Settings and Patients

This retrospective study was approved by the local ethics committee (number 28/GCN-HĐĐĐ). From March 2017 to March 2019, 321 colon cancer patients at any stages were admitted to the University Medical Center at Ho Chi Minh City (UMC HCMC). Among these, we recruited all 43 patients (13.4%) with T4b colon cancer. Inclusion criteria included pathologically confirmed colon adenocarcinoma (>15 cm from the anal verge), radiologic T4b by a CT scan, intraoperative T4b, no metastasis, and Karnofsky score of 0-1. We excluded those who had ever undergone laparotomy surgery, had tumors invading the ureter and duodenum, and had synchronous diseases, recurrent colon cancer, complicated colon cancer (obstruction, perforation, and bleeding), metastatic colon cancer, inflammatory bowel disease, a history of hereditary nonpolyposis colorectal cancer (HNPCC), and familial adenomatous polyposis. We also excluded patients who had involved organs that required major reconstruction (Whipple procedures, extended pelvic exenteration, or ureteral reconstruction). All patients were evaluated before the surgery including physical examination, biopsy colonoscopy, abdominal-pelvic CT scan (preoperative evaluation of tumor depth or adjacent organ invasion involving total colonoscopy (unless obstruction was present) and CT), carcinoembryonic antigen (CEA), and preoperative laboratory testing. The results were discussed in a multidisciplinary meeting before treatment initiation. Patients with nonmetastatic colon cancer did not receive neoadjuvant chemotherapy. Patients were followed up at 1 month after discharge and then every 3 months for the first two years and every six months for the next three years.

### 2.2. Operative Procedures

All laparoscopic surgeries were radical resection with Complete Mesocolic Excision (CME) principles, central vascular ligation (CVL) [26], and en bloc multivisceral resection. Involved adjacent structures were resected en bloc with a surgical margin of 1 cm. This procedure was performed by a single colorectal team with extensive experience in the field of laparoscopic colorectal surgery. The technique included a medial to lateral approach followed by a lateral to medial approach. The specimens were extracted through midline small incision extending the umbilical wound which was then shielded using a wound protector (handmade with a suction tube and surgical gloves). The distal and proximal margins were 5 cm.

### 2.3. Study Outcomes

Preoperative data included age, sex, American Society of Anesthesiologists (ASA) score, tumor location, tumor diameter, differentiation, clinical TNM stage, and CEA level. Operative procedure, anastomosis, operating time, blood loss, conversion, number of lymph nodes harvested, lymph node ratio, and radial resection margin were collected during the surgery. Blood loss was measured by the amount of fluid in the drain minus the amount of lavage. Each gauze removed was considered equivalent to 3 ml of blood loss. All patients were followed 30 days after the surgery, and information about the first flatus, length of hospital stay, iatrogenic complications, postoperative morbidity, mortality, and adjuvant chemotherapy local recurrence, trocar site recurrence, and distant metastasis events was collected. Conversion was defined as unplanned laparotomy. Postoperative morbidity and mortality were defined as events occurring during the hospital stay and within 30 days after the surgery. Postoperative morbidity was also identified based on Clavien-Dindo grade 2 or higher including wound infection, anastomotic leakage, intra-abdominal abscess, ileus, and pneumonia [27]. R1 was identified when tumor cells were present in the pathological resection margin from microscopic assessment.

### 2.4. Data Analysis

The SPSS™ software package (SPSS Inc., Chicago, IL) was used for all statistical analyses. We described the data using frequency and percentage for qualitative measurements and mean and standard deviation for quantitative measurements. Since the data were sparse, we categorized quantitative outcomes based on the cutoff used in previous studies or standard clinical practices including operation time (≤180 min, >180 min), blood loss (<50 ml, 50–100 ml, and >100 ml), time until the first flatus (1–2 days, >2 days), hospital stay (≤7 days, >7 days), and number of lymph nodes harvested (<12, ≥12). Chi-square and Fisher's exact tests were used when appropriate to compare these outcomes in different subgroups. Significance level was used at 5%. The overall survival (OS) and disease-free survival (DFS) rates were calculated using the Kaplan-Meier survival analysis.

## 3. Results

Thirty patients were diagnosed with cT4b, and thirteen were confirmed to have T4b during surgery. Demographic and clinical characteristics of all 43 patients are summarized in Table 1. The mean age was 56.4 (SD = 15.1) years. The majority of patients were male (55.8%). Tumors were identified at the cecum in 4 patients (9.3%), ascending colon in 7 patients (16.3%), hepatic flexure in 1 patient (2.3%), transverse colon in 5 patients (11.6%), splenic flexure in 1 patient (2.3%), descending colon in 14 patients (32.6%), and sigmoid colon in 11 patients (25.6%). Right-sided colon cancer was present in 14 patients (32.6%) and left-sided colon cancer in 29 patients (67.4%). The mean tumor diameter was 7.4 (SD = 2.9) cm. Most of invasive organs were the abdominal wall, intestine, and mesenterium. There were twelve patients having invasion in more than 2 structures. The mean CEA level was 27.3 (SD = 63.6) ng/ml.

The mean operating time was 187, ranging from 80 to 310 minutes, and the mean blood loss was 64.3 ml, ranging from 5 ml to 200 ml. Operation time was significantly associated with CEA level (*p* = 0.011). The blood loss was significantly associated with invaded structures (*p* = 0.013) (Table 2).

The median time until the first flatus was 3 days (range 2–5 days), and the median hospital stay was 7.1 (range 6–11) days (Table 3). The first flatus time and length of hospital stay were marginally associated with CEA level (*p* = 0.062) and ASA score (*p* = 0.085), respectively. There was 1 patient (2.3%) who required conversion due to the invasion of the tumor to the abdominal wall and gonadal vessels and major bleeding. This patient also had leakage at the fifth day after surgery. The overall morbidity rate was 14% (6 cases). Among these, there were 4 cases with wound infection, 1 case with parastomal hernia, and 1 case with anastomotic leakage. As shown in Table 4, the morbidity was marginally associated with the ASA score (*p* = 0.067). There was no mortality at 30 days postoperatively, and one patient had iatrogenic complication (2.3%). This patient had ileum perforation due to dissection, and it is closed by laparoscopically sewing by hand without postoperative morbidity.

The median number of lymph nodes harvested was 13 (IQR = 12-18), and 33 patients (76.7%) had at least 12 lymph nodes harvested. A total of 21 patients (48.8%) had lymph node metastases, and the median lymph node metastasis is 14 (IQR 12-21). There were 3 cases with 11, 2 cases with 10, 2 cases with 7, 2 cases with 6, and 1 case with 4 lymph nodes harvested of the other 10 patients. The radial resection margin was R0 in all 43 patients (100%). The number of harvested lymph nodes was significantly associated with CEA level (*p* = 0.040) and tumor diameter (*p* = 0.005).

**Figure 1:**
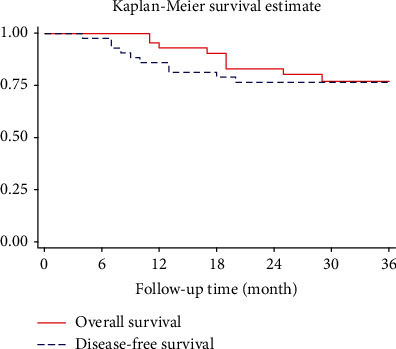
Overall survival and disease-free survival.

In terms of oncologic results, patients were followed up for a mean duration of 31 months (range, 11–48 months). All patients underwent postoperative adjuvant chemotherapy because of advanced stages. No trocar site recurrence was observed during the follow-up. The overall local recurrence rate was 4.8%. The distant metastasis was 18.6%. Among metastasis patients, there were 4 cases of liver (9.3%), 2 cases of lung (4.7%), 1 case of peritoneal (2.3%), and 1 case of liver and peritoneal metastases. The 3-year OS and DFS were 77.3% and 76.6%, respectively (Figure 1).

## 4. Discussion

Laparoscopic colectomy has become a standard treatment for colon cancer because of the advantages in meticulous dissection and faster recovery. However, laparoscopic MVR for T4b colon cancer has not been widely accepted due to the potential of a high conversion rate and increased risk of R1 margins [14, 28]. In our study, we found that laparoscopic surgery for patients with T4b colon cancer might be a safe procedure with both positive surgery indicators and short-term outcomes. For example, our operation time was reasonable and relatively low (187 min) as compared to those in previous studies by Miyake et al. [29] (247 min) and by Takahashi et al. [16] (283.5 min). Moreover, we found lower level of blood loss (64.3 ml) than that reported by Miyake et al. [29] (80 ml) and by Takahashi et al. [16] (57.5 ml); lower number of patients required conversion to open surgery (2.3% compared to 28% and 12.5%). The level of postoperative morbidities was also lower in our study as compared to the study by Miyake et al. [29] (14% and 28.0%). Moreover, in comparison with the study by Takahashi et al. [16], the length of hospital stay in our study was relatively shorter (7.1 days vs. 14 days). Similar to the previous study, we had no death after 30 days [16, 29]. Our positive findings might be due to several reasons from both the patients and the surgeons. In this study, we excluded patients who had tumors invading the ureter (*n* = 14 including 9 cases in the left ureter and 5 cases in the right ureter) because open surgery was indicated. Seven patients with duodenum metastasis were also excluded since these patients might have required major reconstruction of the ureter or Whipple procedures with potential high probability of having postoperative complications [16]. Due to the technical difficulty of procedures used in our study, the involvement of experienced surgeons and a single colorectal team might also contribute to the consistency of the procedure, surgical quality, and thus low level of complication.

Pathological findings are the most important factors to assess the feasibility of surgery in colon cancers. In the present study, there was a satisfactory median number of lymph nodes harvested which is 13. But only more than three-fourths of these patients (76.7%) had at least 12 lymph nodes which is slightly lower than that in the study by Takahashi et al. (83.3%) [16]. The remaining 23.3% had less than 12 lymph nodes, but a total of 43 cases underwent standardized surgical techniques including high ligation, D2 lymphadenectomy, and the proximal and distal margin at least 5 cm from the edge of the tumor. This suboptimal cancer resection was chosen because at that time lymph node harvesting progress had not been standardized and lymph node dissecting was conducted by hand and naked eyes. Moreover, we did not have xylene to dissolve the fat tissue inside the mesentery. Further, in terms of the oncology outcome and survival, R0 resection is essential. However, this might not be achieved by a laparoscopic approach [30]. In our study, R0 resection was observed in all patients, while Takahashi et al. [16] reported 1 case with R1 resection. In a study using a large national database in the US [31], Elnahas et al. reported that patients who underwent a laparoscopic approach did not have a significantly higher positive margin rate than those who underwent open surgery (26.2% and 24.3%, *p* = 0.540). In a systematic review, the R0 resection rate was 91% in the laparoscopic group versus 93% in the open group [32]. Further, Takahashi et al. [16] supposed that the R1 rate is not higher in the laparoscopic group as compared to the open surgery group and R1 has no adverse impact on the laparoscopic approach. The COLOR trial [33] reported the rate of R1 resection in 20% of T4 tumors compared to only 1% of T3 tumors. With such positive result in the resection margin, our overall local recurrence rate was 4.8% while in previous studies, it was 7.7% and 3.1% [34, 35]. In our study, the distant metastases were relatively high with 18.6% and appeared early after surgery (less than 13 months). Among these, most of the cases were liver and lung metastases. There were a case of peritoneal metastasis and a case of simultaneous liver and peritoneal metastasis in comparison with a case of peritoneal metastasis in a study of Takahashi et al. [16].

In terms of survival, the 3-year OS and DFS were 77.3% and 76.6%, respectively. This finding is encouraging when compared with a retrospective study by Japanese authors for 85 cases of T4b colon cancer (38 were treated with a laparoscopic approach, and 47 were treated with an open approach) where the 3-year OS and DFS were about 80% and 73.9%, respectively [35]. In some retrospective studies, the 3-year OS and DFS were 82% and 76% [18] and 83% and 62% [34], respectively, but these studies included both T4a and T4b colon cancers, and the proportion of T4b was substantially higher in the open surgery group. In another study, the 3-year OS and DFS for the laparoscopic group were 68.4% and 52.6%, respectively, but there were only 12 cases of colon cancer; the remaining 26 cases were rectal cancer [36]. The 3-year OS was as high as 94% in another study by Takahashi et al. [16] which indicates the benefit of the laparoscopic approach in patients with T4b colon cancer.

There is one main implication from our study. The encouraging results in the present study such as short-term surgical outcome, conversion rate, morbidity, mortality, and pathological outcomes indicated the feasibility and safety of laparoscopic colectomy for T4b colon cancer. Therefore, in developing countries like Vietnam, radical surgery including a laparoscopic approach could be considered a first-line treatment. This may be especially beneficial for settings with a high volume of patients, limited preoperative staging, and insufficient infrastructure for neoadjuvant treatment. In Vietnam, this approach is advantageous because there is a high number of patients with low social-economic status and poor health condition. However, the laparoscopic approach might only be a suitable option for selected patients in the presence of surgeons with extensive experience and skills. Further, laparoscopic surgery for T4b colon cancer requiring multivisceral resection should be applied with caution due to the possibly high level of R1 resection and other complications [32]. The study has several limitations. Our findings were from a retrospective design using data from medical records with no comparison group. Thus, we were unable to conclude the effectiveness of laparoscopic surgery over open surgery. The feasibility and safety of laparoscopic surgery found in the present study encourage us from doing further projects to compare this approach with different approaches including open surgery. Moreover, this study was performed at a single center with relatively small sample size and a relatively short follow-up period. This might affect the representativeness and the understanding of long-term outcomes such as 5-year survival. Further studies are needed to address these issues.

## 5. Conclusions

Laparoscopic radical colectomy for patients with T4b colon cancer was feasible and safe in selected patients except for those requiring major and complicated reconstruction. This approach could be considered a first-line treatment in the presence of experienced surgeons and fully equipped operating theatre with predefined criteria for patient selection. More multicenter randomized prospective studies are needed to compare this approach with open surgery and to examine its long-term outcomes.

## Figures and Tables

**Table 1:** Demographic and clinical characteristics in patients with T4b colon cancer.

Characteristics	Frequency	Percentage
Sex		
Male	24	55.8
Female	19	44.2
Age (year)		
<50	15	34.9
≥50	28	65.1
ASA		
ASA I	25	58.1
ASA II	18	41.9
Tumor diameter (cm)		
≤4	3	7.0
4-7	20	46.5
≥7	20	46.5
Invaded structure		
Abdominal wall	13	30.2
Intestine, mesenterium	11	25.6
Retroperitoneum	7	16.3
≥2 structures	12	27.9
Differentiation		
Moderate	38	88.4
Poor	5	11.6
CEA level (ng/ml)		
<5	13	30.2
5-99	25	58.1
≥100	5	11.6
Operating procedure		
RC, ERC	14	32.6
LC, ELC	20	46.5
SC, AR	9	20.9

Note: RC: right colectomy; ERC: extended right colectomy; LC: left colectomy; ELC: extended left colectomy; SC: sigmoid colectomy; AR: anterior resection.

**Table 2:** Operation time and blood loss in patients with T4b colon cancer.

Factor	Operation time (min)			Blood loss (ml)			
	≤180 (*n* = 19; 44.2%)	>180 (*n* = 24; 55.8%)	*p*	<50 (*n* = 14; 32.6%)	50-100 (*n* = 15; 34.9%)	>100 (*n* = 14; 32.6%)	*p*
Sex							
Male	9 (37.5)	15 (62.5)	0.321	8 (33.3)	9 (37.5)	7 (29.2)	0.857
Female	10 (52.6)	9 (47.4)		6 (31.6)	6 (31.6)	7 (36.8)	
Age (years)							
<50	6 (40.0)	9 (60.0)	0.686	6 (40.0)	5 (33.3)	4 (26.7)	0.788
≥50	13 (46.4)	15 (53.6)		8 (28.6)	10 (35.7)	10 (35.7)	
ASA							
ASA I	13 (52.0)	12 (48.0)	0.224	10 (40.0)	8 (32.0)	7 (28.0)	0.541
ASA II	6 (33.3)	12 (66.7)		4 (22.2)	7 (38.9)	7 (38.9)	
Tumor diameter (cm)							
≤4	2 (66.7)	1 (33.3)	0.720	1 (33.3)	1 (33.3)	1 (33.3)	0.935
4–7	9 (45.0)	11 (55.0)		7 (35.0)	8 (40.0)	5 (25.0)	
≥7	8 (40.0)	12 (60.0)		6 (30.0)	6 (30.0)	8 (40.0)	
Invaded structure							
Abdominal wall	7 (53.8)	6 (46.2)	0.100	4 (30.8)	3 (23.1)	6 (46.2)	0.013
Intestine, mesenterium	5 (45.5)	6 (54.5)		7 (63.6)	4 (36.4)	0 (0)	
Retroperitoneum	5 (71.4)	2 (28.6)		1 (14.3)	5 (71.4)	1 (14.3)	
≥2 structures	2 (16.7)	10 (83.3)		2 (16.7)	3 (25.0)	7 (58.3)	
Differentiation							
Moderate	16 (42.1)	22 (57.9)	0.640	10 (26.3)	14 (36.8)	14 (36.8)	0.068
Poor	3 (60.0)	2 (40.0)		4 (80.0)	1 (20.0)	0 (0)	
CEA level (ng/ml)							
<5	10 (76.9)	3 (23.1)	0.011	5 (38.5)	7 (53.8)	1 (7.7)	0.056
5-99	7 (28.0)	18 (72.0)		9 (36.0)	6 (24.0)	10 (40.0)	
≥100	2 (40.0)	3 (60.0)		0 (0)	2 (40.0)	3 (60.0)	
Operating procedure							
RC, ERC	8 (57.1)	6 (42.9)	0.482	4 (28.6)	8 (57.1)	2 (14.3)	0.102
LC, ELC	7 (35.0)	13 (65.0)		8 (40.0)	3 (15.0)	9 (45.0)	
SC, AR	4 (44.4)	5 (55.6)		2 (22.2)	4 (44.4)	3 (33.3)	

Note: RC: right colectomy; ERC: extended right colectomy; LC: left colectomy; ELC: extended left colectomy; SC: sigmoid colectomy; AR: anterior resection.

**Table 3:** Description of time of the first flatus and hospitalization according to the patient's clinical features and demographics.

Factor	First flatus time (day)			Hospital stay (day)		
	1-2 (*n* = 14; 32.6%)	>2 (*n* = 29; 67.4%)	*p*	≤7 (*n* = 30; 69.8%)	>7 (*n* = 13; 30.2%)	*p*
Sex						
Male	8 (33.3)	16 (66.7)	0.903	18 (75.0)	6 (25.0)	0.401
Female	6 (31.6)	13 (68.4)		12 (63.2)	7 (36.8)	
Age (year)						
<50	4 (26.7)	11 (73.3)	0.735	12 (80.0)	3 (20.0)	0.487
≥50	10 (35.7)	18 (64.3)		18 (64.3)	10 (35.7)	
ASA						
ASA I	6 (24.0)	19 (76.0.)	0.158	20 (80.0)	5 (20.0)	0.085
ASA II	8 (44.4)	10 (55.6)		10 (55.6)	8 (44.4)	
Tumor diameter (cm)						
≤4	1 (33.3)	2 (66.7)	0.203	2 (66.7)	1 (33.3)	0.999
4-7	9 (45.0)	11 (55.0)		14 (70.0)	6 (30.0)	
≥7	4 (20.0)	16 (80.0)		14 (70.0)	6 (30.0)	
Invaded structure						
Abdominal wall	4 (30.8)	9 (69.2)	0.941	9 (69.2)	4 (30.8)	0.307
Intestine, mesenterium	3 (27.3)	8 (72.7)		10 (90.9)	1 (9.1)	
Retroperitoneum	3 (42.9)	4 (57.1)		4 (57.1)	3 (42.9)	
≥2 structures	4 (33.3)	8 (66.7)		7 (58.3)	5 (41.7)	
Differentiation						
Moderate	13 (34.2)	25 (65.8)	0.999	26 (68.4)	12 (31.6)	0.999
Poor	1 (20.0)	4 (80.0)		4 (80.0)	1 (20.0)	
CEA level (ng/ml)						
<5	4 (30.8)	9 (69.2)	0.062	10 (76.9)	3 (23.1)	0.326
5-99	6 (24.0)	19 (76.0)		18 (72.0)	7 (28.0)	
≥100	4 (80.0)	1 (20.0)		2 (40.0)	3 (60.0)	
Operating procedure						
RC, ERC	5 (35.7)	9 (64.3)	0.999	8 (57.1)	6 (42.9)	0.520
LC, ELC	6 (30.0)	14 (70.0)		15 (75.0)	5 (25.0)	
SC, AR	3 (33.3)	6 (66.7)		7 (77.8)	2 (22.2)	

Note: RC: right colectomy; ERC: extended right colectomy; LC: left colectomy; ELC: extended left colectomy; SC: sigmoid colectomy; AR: anterior resection.

**Table 4:** Description of morbidity and lymph nodes harvested according to the patients' clinical features and demographics.

Factor	Morbidity			Number of lymph nodes harvested		
	Yes (*n* = 6; 14.0%)	No (*n* = 37; 86.0%)	*p*	<12 (*n* = 10; 23.3%)	≥12 (*n* = 33; 76.7%)	*p*
Sex						
Male	3 (12.5)	21 (87.5)	0.999	5 (20.8)	19 (79.2)	0.728
Female	3 (15.8)	16 (84.2)		5 (26.3)	14 (73.7)	
Age (year)						
<50	1 (6.7)	14 (93.3)	0.403	2 (13.3)	13 (86.7)	0.451
≥50	5 (17.9)	23 (82.1)		8 (28.6)	20 (71.4)	
ASA						
ASA I	1 (4.0)	24 (96.0)	0.067	4 (16.0)	21 (84.0)	0.275
ASA II	5 (27.8)	13 (72.2)		6 (33.3)	12 (66.7)	
Tumor diameter (cm)						
≤4	0 (0)	3 (100)	0.787	3 (100)	0 (0)	0.005
4-7	4 (20.0)	16 (80.0)		2 (10.0)	18 (90.0)	
≥7	2 (10.0)	18 (90.0)		5 (25.0)	15 (75.0)	
Invaded structure						
Abdominal wall	2 (15.4)	11 (84.6)	0.377	6 (46.2)	7 (53.8)	0.159
Intestine, mesenterium	0 (0)	11 (100)		2 (18.2)	9 (81.8)	
Retroperitoneum	1 (14.3)	6 (85.7)		1 (14.3)	6 (85.7)	
≥2 structures	3 (25.0)	9 (75.0)		1 (8.3)	11 (91.7)	
Differentiation						
Moderate	6 (15.8)	32 (84.2)	0.999	8 (21.1)	30 (78.9)	0.575
Poor	0 (0)	5 (100)		2 (40.0)	3 (60.0)	
CEA level (ng/ml)						
<5	0 (0)	13 (100)	0.216	4 (30.8)	9 (69.2)	0.040
5-99	5 (20.0)	20 (80.0)		3 (12.0)	22 (88.0)	
≥100	1 (20.0)	4 (80.0)		3 (60.0)	2 (40.0)	
Operating procedure						
RC, ERC	2 (14.3)	12 (85.7)	0.448	2 (14.3)	12 (85.7)	0.653
LC, ELC	4 (20.0)	16 (80.0)		6 (30.0)	14 (70.0)	
SC, AR	0 (0)	9 (100)		2 (22.2)	7 (77.8)	

Note: RC: right colectomy; ERC: extended right colectomy; LC: left colectomy; ELC: extended left colectomy; SC: sigmoid colectomy; AR: anterior resection.

## Data Availability

The data used to support the findings of this study are available from the corresponding author upon request.
